# Supervision and autonomy of ophthalmology residents in the outpatient clinic in the United States II: a survey of senior residents

**DOI:** 10.1186/s12909-019-1620-0

**Published:** 2019-06-13

**Authors:** Eric L. Singman, Michael V. Boland, Jing Tian, Laura K. Green, Divya Srikumaran, The Writing Committee of the Ophthalmology Program Directors’ Study Group (OPDSG), Andrew J. Barkmeier, Andrew J. Hendershot, Andrew Thliveris, Daniel B. Moore, Darrell WuDunn, David J. Goldman, Divya Srikumaran, Evan L. Waxman, Gary Domeracki, Gary L. Legault, Jeff Pettey, John J. Chen, Joshua H. Olson, Jules Winokur, Laura K. Green, Marcus Colyer, Martin Mayers, Michael J. Wilkinson, Michael S. Lee, Michael V. Boland, Misha Syed, Mitchell Drucker, Mitchell P. Weikert, Parisa Taravati, Peter Veldman, Pratap Challa, Preston H. Blomquist, R. Michael Siatkowski, Robert Granadier, Shane Havens, Susan M. Culican, Tara A. Uhler, Thomas J. Whittaker

**Affiliations:** 1 General Eye Services, Wilmer Eye Institute, Johns Hopkins School Of Medicine, Wilmer B-29, 600 N. Wolfe St., Johns Hopkins Hospital, Baltimore, MD 21287 USA; 2 Bloomberg School of Public Health, Johns Hopkins University, Baltimore, USA; 3 Lifebridge Health Krieger Eye Institute, Baltimore, USA

**Keywords:** Autonomy, Supervision, Resident, Ophthalmology, Education, Outpatient, Clinic, Professionalism, Stress, Burnout

## Abstract

**Background:**

A balance between autonomy and supervision can be difficult to obtain in medical education. In this study, we sought to determine whether the presence and level of supervision of ophthalmology resident outpatient clinic correlates with metrics of resident success, professionalism and stress.

**Methods:**

A survey was emailed to all US ophthalmology program directors requesting it be forwarded to PGY4 residents. Questions included whether their program provided a resident-hosted outpatient clinic, and if so, whether residents were mandated to discuss every patient with faculty. Residents were assigned to three categories based on this question (0: no clinic, 1: mandated faculty input, 2: discretionary faculty input). Success metrics included numbers of manuscripts submitted, OKAP scores and success in obtaining fellowships. Professionalism metrics included rating comfort obtaining informed consent, breaking bad news, managing time in clinic, and confidence in providing care in various settings. Residents affirming participation in a continuity clinic also provided perceptions of the level of supervision and how the clinic affected stress.

**Results:**

Category 1 residents perceived somewhat too much supervision, while category 2 residents felt that they had somewhat insufficient supervision. The majority of residents in either category did not feel that the continuity clinic affected their overall stress, although those who reported a change in stress usually indicated that the presence of the clinic increased stress. There were no other statistically significant differences between the responses from any category.

**Conclusions:**

The presence of a resident-hosted continuity clinic neither adds nor detracts from the success or sense of professionalism of ophthalmology residents. However, when such a clinic is present, the degree of supervision appears to correlate inversely with resident perception of autonomy. These results suggest that the decision of a training program to offer a clinic hosted by residents offering comprehensive continuity care can be informed primarily by faculty and trainee philosophy and personal preferences without comprising education quality, clinical efficiency, residents’ perception of stress or their success in fellowship matching.

**Electronic supplementary material:**

The online version of this article (10.1186/s12909-019-1620-0) contains supplementary material, which is available to authorized users.

## Background

Balancing autonomy and supervision is both a goal and challenge of graduate medical education [1–8]. In a previous report, we presented the landscape of supervision styles for ophthalmology trainees in the outpatient clinic setting [2]. That study, based upon a survey of program directors, suggested that there was no substantial correlation between the level of supervision in the outpatient setting and metrics such as the number of residents in the program, demographic sites of the clinics, number of faculty or contributions of resident-hosted clinics to overall resident surgery volumes. The current study has sought to determine whether the presence of a resident-hosted continuity clinic, and the degree of faculty supervision therein, might correlate with metrics of programmatic success and resident professionalism. This second study is a joint effort of the Ophthalmology Program Directors Study Group (OPDSG), currently comprised of 31 program directors (i.e., 27% of US training programs). This group formed after the first study because it was felt that the effort to identify evidence-based best practices in resident education deserved widespread support.

## Methods

An 18-question online survey was emailed to all 115 ACGME-accredited ophthalmology residency program directors in April 2018, requesting they forward the survey to all third-year residents in their respective programs. Follow-up emails to all program directors as well as program coordinators were sent in May 2018. To encourage responses, a $10 charitable donation was pledged to Research to Prevent Blindness for every completed survey. All emails included an introductory letter explaining the purpose of the study, providing a link to the previous study [2] and confirming that this study was reviewed and approved by the Johns Hopkins Institutional Review Board (IRB00069769.)

This was an anonymous survey where no personal identifying information was collected. The survey was designed using forced choices, Likert scales, and in some instances, the option to provide additional free-text information. The survey uses simple Boolean logic so that responders could be directed to germane questions based upon their answers while skipping over questions that might be redundant or non-sequitur. The survey as it appeared to respondents can be accessed with this link: https://jhmi.co1.qualtrics.com/jfe/form/SV_80pRNHwoQaXTuiF. Supporting data including the anonymized respondents of those surveyed are available upon request from the corresponding author.

The survey questions and the logic-flow instructions based upon the answers to each question are presented as Additional file 1. The first set of questions were meant to assess the academic success of the residents. The OPDSG queried residents on the number of publications they submitted, their overall percentile on the Ophthalmology Knowledge Assessment Program (OKAP, an in-service exam created by the American Academy of Ophthalmology [AAO] [9]), and their success and motivation in applying for post-graduate fellowships. The second set of survey questions queried resident comfort level with situations requiring a mature level of professionalism including obtaining informed consent, relaying disappointing news to patients, and managing patients in the emergency, inpatient and outpatient setting. The third set of questions were meant to identify whether the residents’ training included participation in a resident-hosted comprehensive continuity clinic. Those responding positively were asked how that clinic was supervised in terms of whether a faculty member was required to discuss, and/or see every patient encountered. The initial survey defined five categories (listed below from lowest to highest) with respect to the degree of resident autonomy:

0. No resident-hosted clinic
1. Resident-hosted clinic where the supervisor must see every resident patient
2. Resident-hosted clinic where the supervisor must discuss every patient with the resident but not necessarily see the patient
3. Resident-hosted clinic where the supervisor was not required to discuss every patient with the resident.
4. Resident-hosted clinic where the supervisor was not onsite but available by telephone for indirect supervision.

However, the number of responses for categories 1 and 4 were considered too low to obtain meaningful statistics so category 1 was combined with category 2 and category 3 was combined with category 4. The new categories are listed below.

0. No resident-hosted clinic
1. Resident-hosted clinic where the supervisor must at least discuss (if not also see) every patient encountered
2. Resident-hosted clinic where the supervisor discussed (and possibly saw) a patient at the resident’s discretion.

The final set of survey questions were directed to residents identifying with (initial) categories 1–4, (i.e., new categories 1 and 2) exploring how they perceived the degree of supervision they were provided and whether the clinic experience affected their levels of stress.

The finalized categories of resident clinics were assigned an ordinal score of 0–2 to explore whether statistical correlations existed between the presence of a resident-hosted clinic (and if so, the level of supervision) and the other metrics gleaned in the survey.

For the sample size calculation, we determine that we required at least 97 complete response to obtain a marginal error of 5% and confident interval of 95%. We employed the ANOVA test for the questions exploring numbers of submissions for publication, OKAP score, rank of matched fellowship program, comfort breaking bad news, comfort obtaining informed consent, numbers of patients seen daily in clinic and confidence managing patients in the outpatient setting, emergency department and inpatient setting. The chi squared test was employed for the questions pertaining to whether a resident applied and matched to a post-graduate fellowship and whether they felt that the outpatient continuity clinic affected their level of stress during training. Finally, the Fisher Exact test was employed for the questions concerning difficulty managing time in the clinic and whether the outpatient clinic increased or decreased the level of stress experienced during training.

## Results

There were 116 completed responses: 39, 29 and 48 in categories 0, 1 and 2, respectively. Estimating that there are approximately 490 senior ophthalmology residents in the United States, this represents a response rate of 24%. The average number of submissions for publications, OKAP overall percentile, rate of application for fellowship, motivation therein, and success and rank of program if matched are provided in Table 1. There was no statistically significant correlation between these metrics and the category of resident outpatient clinic.

**Table 1:** The average number of submissions for publication by residents, their overall percentile on the most recent OKAP examination, the percentage of respondents who applied for fellowship, the percentage of applicants matching and where they ranked the matching program, and the percentages of applicants who applied because of great interest, desire to enhance employment prospects and desire to obtain more training in order to be comfortable entering practice (multiple responses were permitted) for clinic categories 0 (no resident-hosted clinic), 1 (resident-hosted clinic where faculty must discuss and/or see every patient encountered) and 2 (resident-hosted clinic where faculty see patients at the residents’ discretion and the faculty member may or may not be on-site)

Clinic Category	Average # submissions for publication (95% CI)	Percentile OKAP (95% CI)	% Applied for fellowship (95% CI)	% Accepted for fellowship (95% CI)	Average Rank of matched program (95% CI)	% Great interest in specialty (95% CI)	% Enhance employment prospects (95% CI)	% Not yet comfortable entering practice (95% CI)
0 (no clinic)	3.5 (2.5–4.5)	62 (53–71)	67 (51–79)	92 (76–98)	3rd (1–4)	100 (87–100)	31 (50–84)	8 (2–24)
1 (must discuss)	3.5 (2.0–5.0)	51 (40–62)	62 (44–77)	100 (82–100)	3rd (1–4)	100 (82–100)	39 (20–61)	6 (1–25)
2 (resident discretion)	5.1 (3.2–6.9)	64 (56–71)	75 (61–85)	100 (90–100)	2nd (1–3)	89 (75–96)	42 (27–58)	3 (0.5–14)
*p*-value	0.23	0.12	0.46	0.12	0.40	0.38	0.69	0.81
Test used to calculate *P*-value	ANOVA	ANOVA	Chi-square	Chi-square	ANOVA	Fisher exact	Fisher exact	Fisher exact

Each metric is followed by the 95% CI. The test used to calculate the whether there was statistically significant differences in the metrics is provided

Table 2 provides the data for the resident scoring concerning their comfort breaking bad news and obtaining informed consent, their difficulty managing time in the outpatient clinic and the number of clinic patients they see on a typical day, and their confidence managing patients in the clinic and providing consultations in the emergency department and inpatient wards. There was no statistically significant correlation between these metrics and the category of resident outpatient clinic.

**Table 2:** For clinic categories 0 (no resident-hosted clinic), 1 (resident-hosted clinic where faculty must discuss and/or see every patient encountered) and 2 (resident-hosted clinic where faculty see patients at the residents’ discretion and the faculty member may or may not be on-site), this table shows the residents’ comfort level breaking bad news to a patient and obtaining informed consent (1 = extremely comfortable, 2 = somewhat comfortable, 3 = neither comfortable nor uncomfortable, 4 = somewhat uncomfortable, 5 = extremely uncomfortable), perceived difficulty managing time in clinic (1 = very difficult, 2 = moderate difficulty, 3 = not much difficulty), average number of patients seen in a typical day, and confidence managing patients in clinic and providing consultations in the emergency department and inpatient wards (1 = very confident, 2 = somewhat confident, 3 = neutral, 4 = somewhat diffident, 5 = very diffident)

Clinic Category	Comfort giving bad news	Comfort obtaining informed consent	Difficulty managing time in clinic	Average number of clinic patients daily	Confidence managing patients in clinic	Confidence offering consults in ED and ward
0	1.8 (1.6–2.1)	1.2 (1.0–1.5)	2.0 (1.8–2.2)	20 (17–22)	1.6 (1.3–1.9)	1.4 (1.2–1.5)
1	1.5 (1.3–1.7)	1.1 (1.0–1.2)	1.9 (1.6–2.0)	20 (18–22)	1.6 (1.2–1.7)	1.2 (1.1–1.4)
2	1.8 (1.6–2.1)	1.1 91.0–1.2)	1.8 (1.6–1.9)	21 (19–23)	1.3 (1.2–1.5)	1.3 (1.1–1.4)
*p*-value	0.15	0.49	0.21	0.73	0.21	0.51
Test used to calculate *P*-value	ANOVA	ANOVA	ANOVA	ANOVA	ANOVA	ANOVA

Each metric is followed by the 95% CI. The *p*-value refers to the difference between the metrics in any given column

For those residents in clinic category 1 and 2 (i.e., in programs providing a resident-hosted outpatient comprehensive continuity clinic), the average levels of perceived supervision and effect on overall stress during training is presented in Table 3. No resident felt that there was far too much or far too little supervision. Residents with category 1 clinics felt there was moderately more than enough supervision while residents in category 2 sensed there was somewhat insufficient supervision and this difference was statistically significant (*p* < .0001). Of the residents who felt that the continuity clinic affected their stress during training, the great majority in both categories felt that the experience increased stress. Notably, while it appears that residents in category 2 felt their clinics contributed to stress more often than those in category 1, this difference did not reach statistical significance (*p* = .11). Concerning the increased stress reported, it should be noted that of their 12 free-text comments, 7 were negative while 5 were positive. Positive comments included “good stress promotes learning”, “relationships with known patients alleviated stress”, “stress is appropriate as I care about my own patients’ outcomes”, “increased stress is not necessarily a bad thing” and stress “was helpful preparing for real practice”.

**Table 3:** For clinic categories 1 (resident-hosted clinic where faculty must discuss and/or see every patient encountered) and 2 (resident-hosted clinic where faculty see patients at the residents’ discretion and the faculty member may or may not be on-site), this table shows how residents felt about the degree of supervision provided in their continuity clinics (1 = moderately too much, 2 = just right, 3 = moderately insufficient), as well as whether the continuity clinic experience affected the overall stress level and if so, in what way

Clinic Category	Perceived degree of supervision	Did continuity clinic affect stress?	Increased stress	Decreased stress
1	1.7 (1.5–1.9)	28% (15–46%)	100% (68–100%)	0% (0–32%)
2	2.2 (2.0–2.3)	46% (33–60%)	91% (72–97%)	9% (3–28%)
*p*-value	< 0.0001	0.11	0.53	0.53
Test used to calculate *P*-value	t-test	Chi-square	Fisher exact	Fisher exact

The 95% CI is provided after each metric. The *p*-value refers to whether there is a difference between the metrics in any given column

## Discussion

As in our previous study [2], we did not find significant correlations between the presence of a resident-hosted continuity clinic or, when such a clinic was present, the level of supervision offered therein, and the various metrics of resident success explored. These metrics included the number of resident manuscripts submitted, OKAP scores, and application and success rates of obtaining fellowships. One might suggest that a greater number of completed surveys might have permitted the wider granularity in the categories of training environments described in our previous study, thus revealing significant differences. In addition, since residents volunteered to participate, the authors could not control for negative or positive biases of respondents. Furthermore, one could suggest employing different metrics of resident success than the ones chosen. Presently, there are no established or generally accepted metrics of this type; there may be utility for a governing body such as the Accreditation Council for Graduate Medical Education to consider creating such a list.

Aside from metrics of success, we also explored metrics related to professionalism and resident perceptions thereof, such as comfort obtaining informed consent, relaying disappointing news to patients, and managing patients in the emergency, inpatient and outpatient setting. Patient volumes were similar for all clinic styles, suggesting there is no apparent effect on clinical efficiency or the quantity of outpatient educational opportunities inherent in every encounter. Furthermore, fewer than half the residents serving in their own continuity clinics felt that this teaching environment affected overall stress. In addition, even though perceived stress was increased almost uniformly when there was a reported effect, this stress was not always considered to be negative. This was borne out by the responses to the free-text questions which supported the idea that the residents felt that the clinic was a good learning opportunity and that they appreciated having ownership of their patients.

Further research in this area should include surveying young post-graduates, querying them on metrics such as their professional setting (e.g., academic, research, industry, private practice), application- and pass-rate as well as number of attempts for the American Board of Ophthalmology Written Qualifying- and Oral-Examinations, and perception of how well their outpatient experiences prepared them for practice. The authors conclude that residency training programs can make decisions about offering resident-hosted continuity clinics with equanimity.

This key result of the present study could have important implications for residents as well as training programs. For example, increased autonomy has been correlated with lower levels of resident burn-out [10–12], a significant and increasingly recognized concern of training [13–16]. It may be that residents prone to burnout would prefer a training program with opportunities to gain a sense of greater autonomy such as that outpatient clinic where they take ownership of patients and have discretion concerning supervisor input. Alternatively, residents who are more stress- or risk-averse might prefer an outpatient setting with closer supervision; it has been reported that a resident’s perception of insufficient oversight was correlated with other negative perceptions of clinical training, such as increased stress and risk of medical errors [5].

Whereas residents must be focused on learning, residency programs must take other aspects of clinical operations into account. For example, a program might require supervisors to corroborate (and bill for) every resident encounter to ensure the economic viability of the resident clinic and ensure positive margins for supervisors in the resident clinic. The present study suggests that this arrangement would not reduce resident success or their sense of professionalism. Indeed, such an arrangement might even reduce medical errors [17–19], although there is debate on this issue [20]. On the other hand, in situations where 100% sign-off of charts has no financial advantage, then the perceived diminution to the residents’ sense of autonomy may rise in prioritization. Furthermore, one could suggest that if programs employed preceptors in the clinic with a variety of supervision styles, there is no evidence that this would diminish the educational or professional training.

In conclusion, the present study suggests that the decision of a training program to offer a clinic hosted by residents offering comprehensive continuity care can be informed primarily by faculty and trainee philosophy and personal preferences without comprising education quality, clinical efficiency, residents’ perception of stress or their success in fellowship matching.

## Conclusions

The presence of a resident-hosted continuity clinic neither adds nor detracts from an ophthalmology resident’s success (as measured by number of resident manuscripts submitted, OKAP scores, and application and success rates of obtaining fellowships) or sense of professionalism (as measured by self-reported comfort in obtaining informed consent, relaying disappointing news to patients, and managing patients in the emergency, inpatient and outpatient setting). However, when such a clinic is present, the degree of supervision appears to correlate inversely with resident perception of autonomy. These results suggest that the decision of a training program to offer a clinic hosted by residents offering comprehensive continuity care can be informed primarily by faculty and trainee philosophy and personal preferences without comprising education quality, clinical efficiency, residents’ perception of stress or their success in fellowship matching.

## Additional file

Additional file 1: The survey in the format that the residents experienced. (DOCX 18 kb)

## Data Availability

Anonymized data is available from the corresponding author upon request.

## Abbreviations

AAO: American Academy of Ophthalmology
ACGME: Accreditation Council for Graduate Medical Education
OKAP: Ophthalmology Knowledge Assessment Program
OPDSG: Ophthalmology Program Directors’ Study Group
PGY4: Post Graduate Year 4
