# Supplementary material for: Supervision and autonomy of ophthalmology residents in the outpatient clinic in the United States II: a survey of senior residents
Source: BMC Med Educ. 2019 Jun 13;19:202. doi: 10.1186/s12909-019-1620-0 (PMC6567568; doi:10.1186/s12909-019-1620-0)
Supplement: Supplementary file 1 — The survey in the format that the residents experienced. (DOCX 18 kb) [file 12909_2019_1620_MOESM1_ESM.docx]

Supplement 1. The survey in the format that the residents experienced.

Supervision and Autonomy II: 3rd year residents

Start of Block: Block 1

Q1 How many articles have you submitted for publication (regardless of whether the article was accepted, and this includes manuscripts, case reports, book chapters and reviews)

_______ # articles

Q2 What was the percentile of your overall score in your 3rd Year (i.e., this academic year) OKAP examination?

_______ % overall OKAP score

Q3 Have you applied for a post Graduate Fellowship Training Program?

- Yes
- No

Skip To: End of Block If Q3 = No

Q4 Did you match into a post Graduate Fellowship Training Program?

- Yes
- No

Skip To: Q6 If Q4 = No

Q5 Where did you rank the Fellowship Training Program into which you matched?

_______ Rank location

Q6 Why did you decide to apply for post graduate Fellowship training (**choose all that apply**)

- I was greatly interested in the specialty training
- I felt that the additional training would enhance my chances of finding employment
- I felt that I needed the additional training because I did not yet feel comfortable entering practice
- Other (please free-text any other reason you decided to apply for Fellowship training ________________________________________________

End of Block: Block 1

Start of Block: Block 3

Q7 How comfortable are you breaking bad news (e.g., poor outcome or medical mistake) to a patient?

- Extremely comfortable
- Somewhat comfortable
- Neither comfortable nor uncomfortable
- Somewhat uncomfortable
- Extremely uncomfortable

Q8 How comfortable are you obtaining informed consent from a patient for a procedure?

- Extremely comfortable
- Somewhat comfortable
- Neither comfortable nor uncomfortable
- Somewhat uncomfortable
- Extremely uncomfortable

End of Block: Block 3

Start of Block: Block 4

Q9 How much difficulty did you have in time management of your clinic?

- A great deal; It is hard to see all my patients and finish on time
- A moderate amount; I usually finish on time but feel rushed
- Not much, I usually finish on time

Q10 How many patients each day do you see in your own clinic?

|  | 0 | 5 | 10 | 15 | 20 | 25 | 30 | 35 | 40 | 45 | 50 |
| --- | --- | --- | --- | --- | --- | --- | --- | --- | --- | --- | --- |

|  | 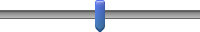 |
| --- | --- |

Q11 How confident do you feel managing patients in your own clinic?

- Very confident
- somewhat confident
- neutral
- somewhat diffident
- very diffident

Q12 How confident do you feel performing inpatient and Emergency Department consultations?

- Very confident
- Somewhat confident
- Neutral
- Somewhat diffident
- Very diffident

End of Block: Block 4

Start of Block: Block 2

Q13 Does your program provide an outpatient clinic where residents provide continuity care to their own comprehensive ophthalmology patients with or without direct supervision?   **NOTE: If your program has more than 1 clinic where residents provide continuity care to their own comprehensive patients, please have in mind the clinic where you spent the most time during your residency program or the clinic where you saw the majority of your own comprehensive patients.
If you answer NO you will jump to End of Survey.**

- Yes
- No

Skip To: End of Survey If Q13 = No

End of Block: Block 2

Start of Block: Block 1

Q14 In your continuity clinic, must you discuss every patient you treat with a supervising faculty member?

- YES and that patient must also be seen by the faculty member who will co-sign the documentation of the encounter
- YES but that patient might not necessarily be seen by the faculty member
- NO, the supervising faculty member is available to provide direct or indirect supervision, but only at the resident's discretion and request
- NO, the faculty member is not directly in the clinic but is available by phone for indirect supervision at the resident's discretion and request

Q15 How do you feel about the level of faculty supervision you were provided in your continuity clinic?

- Far too much
- moderately too much
- just right
- moderately insufficient
- very insufficient

Q16 Do you feel your continuity outpatient clinic experience affected the level of stress you perceived during your resident?

- Yes
- No

Skip To: End of Survey If Q16 = No

Q17 Do you feel your continuity clinic reduced or increased the level of stress you experienced during residency?

- Increased level of stress
- Reduced level of stress

Q18 Please explain (free text)  the way(s) in which your continuity clinic affected the stress you experienced in residency.

________________________________________________________________

End of Block: Block 1
